# Supplementary material for: Si-Wu Water Extracts Protect against Colonic Mucus Barrier Damage by Regulating Muc2 Mucin Expression in Mice Fed a High-Fat Diet
Source: Foods. 2022 Aug 18;11(16):2499. doi: 10.3390/foods11162499 (PMC9407452; doi:10.3390/foods11162499)
Supplement: Supplementary file 1 [file foods-11-02499-s001.zip › foods-1804337-supplementary.pdf]

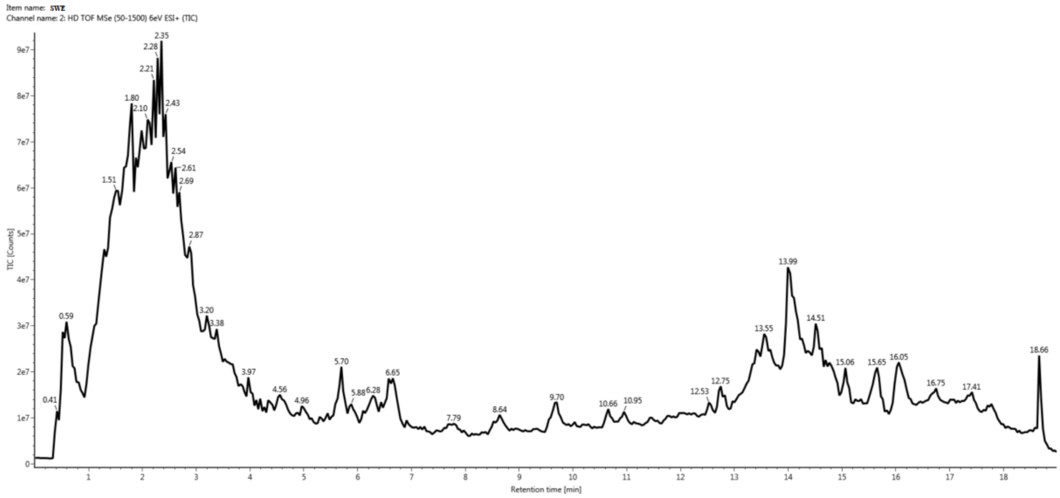

Figure S1. Total ion chromatogram of SWE in positive ion mode.

Table S1. Ingredients and Composition of Different Diets

	**CON**	**HFD**
**Ingredient (g)**		
Casein, 30 Mesh	200	200
L-Cystine	3	3
Corn starch	506.2	0
Maltodextrin 10	125	125
Sucrose	68.8	68.8
Cellulose, BW200	50.0	50.0
Soybean Oil	25.0	25.0
Lard	20.0	245.0
Mineral Mix S10026	10.0	10
DiCalcium Phosphate	13.0	13
Calcium Carbonate	5.5	5.5
Potassium Citrate, 1 H2O	16.5	16.5
Vitamin Mix V10001	10.0	10.0
Choline Bitartrate	2.00	2.0
FD&C Yellow Dye#5	0.04	0
FD&C Blue Dye#1	0.01	0.05
Total	1055.05	773.85
**Composition**		
Protein (g/100g)	19.2	26.2

	CON	HFD
TEP[a] (%)	20	20.0
Fat (g/kg)	4.3	34.9
TEF[b] (%)	10.0	60.0
Carbohydrate (g/kg)	67.3	26.3
TEC[c] (%)	70.0	20.0
**total energy(kcal/g)**	**3.85**	5.24

[a]TEP, total energy from protein.
[b]TEF, total energy from fat.
[c]TEC, total energy from carbohydrate.

Table S2. Compounds identified in SWE by UPLC-Q/TOF-MS in positive ion mode.

Identified components	Mass error (ppm)	Observed RT (min)	Response area	Relative area (%)
5-HMF	12.5	1.45	9270	0.30%
Catalpol	-7	1.04	11501	0.38%
Chlorogenic acid	0	1.46	421804	13.77%
Ligustilide	0.2	6.65	434358	14.18%
Paeoniflorin	1.7	2.34	3062303	100.00%
Verbascoside	1.3	2	354552	11.58%
